# Complement Inhibition in Geographic Atrophy: Clinical Evidence for Pegcetacoplan and Avacincaptad Pegol

**DOI:** 10.7759/cureus.109282

**Published:** 2026-05-20

**Authors:** Gloriana Orozco Loaiza, Maria Jimena Alfaro Guerra, Adrián Murillo Sotela, Paula Villalobos Villalobos

**Affiliations:** 1 Faculty of Medicine, Universidad de Costa Rica, San José, CRI

**Keywords:** age-related macular degeneration (amd), avacincaptad pegol, complement c3 inhibition, complement c5 inhibition, complement system, geographic atrophy (ga), pegcetacoplan

## Abstract

Geographic atrophy (GA) represents the advanced form of non-neovascular age-related macular degeneration (AMD) and is associated with progressive and irreversible vision loss. Evidence has implicated the dysregulation of the complement cascade as a pivotal factor in GA pathophysiology, leading to the development of complement inhibition therapies. Pegcetacoplan, a C3 inhibitor, and avacincaptad pegol, a C5 inhibitor, are currently the only therapies approved by the Food and Drug Administration (FDA) for GA secondary to AMD. Major clinical trials, including OAKS, DERBY, GALE, GATHER1, and GATHER2, have demonstrated a statistically significant reduction in GA lesion area, with reductions of approximately 16-22% for pegcetacoplan and 14-28% for avacincaptad pegol compared with sham. Despite favorable structural outcomes, functional endpoints like best-corrected visual acuity (BCVA) and low-luminance visual acuity (LLVA) have shown limited improvement, and concerns remain regarding their side effect profile. This narrative review provides an overview of GA, emphasizing its pathophysiology and the role of the complement cascade, and discusses the clinical relevance of complement inhibitors in the current management of the disease.

## Introduction and background

Age-related macular degeneration (AMD) is a common retinal disease characterized by a progressive degeneration of the central retina that can result in irreversible vision loss [1]. It represents one of the leading causes of vision loss in individuals over 55 years of age in developed countries [2]. As global life expectancy continues to increase, the prevalence of AMD is expected to rise, with projections estimating that approximately 288 million individuals worldwide may be affected by 2040 [2]. 

Geographic atrophy (GA) represents the advanced stage of non-neovascular (dry) AMD and is characterized by retinal lesions that gradually expand and can ultimately lead to central vision loss [3]. The prevalence of GA increases markedly with age and has been reported to affect up to one in five individuals aged 85 years or older in at least one eye [4,5]. As populations continue to age globally, the incidence of GA is expected to increase in the coming decades [4,5].

Progressive loss of central vision significantly affects daily activities such as reading, driving, and recognizing faces, often leading to permanent vision impairment and reduced independence and quality of life [6]. Despite its clinical significance, therapeutic options for GA have historically been limited. Management strategies have traditionally focused on conservative measures, including smoking cessation and Age-Related Eye Disease Study (AREDS)-based nutritional supplementation, with the goal of slowing disease progression in earlier stages [7]. However, these strategies do not prevent or reverse retinal atrophy once GA has developed, highlighting the ongoing unmet clinical need for disease-modifying therapies [8].

In recent years, dysregulation of the complement cascade has been strongly implicated as a key contributor to the pathogenesis of GA. This has prompted increasing interest in complement proteins as potential therapeutic targets. In 2023, the United States Food and Drug Administration (US FDA) approved the first two pharmacologic therapies for GA secondary to AMD, both complement inhibitors. Pegcetacoplan was approved in February 2023 and avacincaptad pegol in August 2023 [9].

This narrative review summarizes key aspects of GA, including its pathophysiology and the role of the complement system in this disease. It also reviews the key clinical evidence supporting the complement inhibition therapies, focusing on pegcetacoplan and avacincaptad pegol, and discusses their relevance in the current management of GA. This review focuses on providing a structured framework to understand GA rather than an exhaustive summary of clinical trial data. 

## Review

Methods

Literature Search Strategy

This narrative review was developed through a comprehensive literature search conducted between December 2025 and May 2026, using several scientific databases including PubMed Central and Scopus, complemented by manual searches of selected journals such as BMC Ophthalmology. Additional sources were identified through reputable online platforms such as the American Academy of Ophthalmology and ClinicalTrials.gov. Grey literature sources were also reviewed, including conference presentations from the Annual Meeting of the Retina Society. The search included articles between January 2016 and May 2026. Additional relevant studies identified during the manuscript revision process were incorporated to ensure that the review reflected the most current available evidence.

Key Words and Search Terms

The search strategy included the terms "geographic atrophy", "age-related macular degeneration", "dry AMD", "complement cascade", "complement inhibitor", "C3 inhibitor", "C5 inhibitor", "pegcetacoplan", and "avacincaptad pegol", in addition to the names of major clinical trials such as "OAKS", "DERBY", "GALE", "GATHER1", and "GATHER2". Search terms were combined using Boolean operators (AND/OR) to enhance the reproducibility of the literature selection process.

Inclusion and Exclusion Criteria

Eligible publications included clinical trials, observational studies, and prior review articles addressing the pathophysiology of GA, the role of the complement cascade, and therapeutic strategies targeting complement inhibition. Particular emphasis was placed on studies evaluating the efficacy and safety of pegcetacoplan and avacincaptad pegol. 

Only articles published in English were included, which may have introduced language bias and limited the inclusion of potentially relevant studies published in other languages. Publications that did not directly address the pathophysiology of GA, complement system involvement, or complement-targeted therapies were excluded. 

Study Selection and Review Approach

The study selection process involved an initial screening of titles and abstracts to assess relevance. Articles meeting the inclusion criteria underwent full-text review to determine final inclusion based on the predefined inclusion criteria. Reference lists of selected articles were manually reviewed to identify additional relevant studies. 

A total of 60 articles and two conference presentations were initially identified. After the removal of duplicate data, 58 articles and two conference presentations underwent screening. After the application of the eligibility criteria, 56 articles and one conference presentation were included in the final review.

As this work was designed as a narrative review rather than a formal systematic review, article selection was guided primarily by conceptual and clinical relevance to the topic rather than by a formal systematic review protocol. This approach allowed a clinically focused discussion and synthesis of the literature regarding GA and complement inhibition therapies. However, it may also result in the omission of some relevant studies. 

No formal meta-analysis or pooled statistical methods were performed, and study findings were summarized descriptively. A formal risk-of-bias assessment tool was not applied. The evidence included was primarily from peer-reviewed articles on clinical trials, extension studies, and observational data evaluating the therapies. However, important limitations of the literature used should be acknowledged, including variability in study design, follow-up duration, patient selection criteria, and outcome measures in trials. The inclusion of conference presentations and real-world data may introduce limitations related to incomplete long-term safety and efficacy assessment. These factors should be considered when interpreting the current evidence presented in this review. 

GA overview

Classification

AMD is clinically classified into three stages based on characteristics involving drusen size and pigmentary abnormalities. Early AMD is defined by the presence of medium-sized drusen (63-125 μm), whereas intermediate AMD is characterized by large drusen (≥125 μm) and pigmentary changes [4]. The advanced stage is further organized into two categories: neovascular AMD (exudative or wet) and non-neovascular AMD (non-exudative or dry) [2,9,10].

Neovascular AMD is characterized by the development of choroidal neovascularization (CNV) under the retina and macula, while non-neovascular AMD results from the progressive loss of photoreceptors and retinal pigment epithelium (RPE), ultimately leading to retinal atrophy [10]. GA represents the advanced form of non-neovascular AMD and is characterized by the progressive loss of RPE cells, photoreceptors, and the choriocapillaris [4,5]. The disease typically begins in the parafoveal region and gradually expands to involve the central fovea, resulting in central vision loss [6].

Prevalence

GA affects an estimate of five million individuals worldwide. In the United States, it affects 0.28% of individuals aged 60-64 and 0.98% of those aged 70-74 [11]. In Nordic countries, its prevalence ranges from 0.4% of individuals aged 60-69 to 7.6% of those aged 80 years or older [12]. Overall, the prevalence increases significantly with age. 

GA is more commonly observed in populations of European ancestry compared with Asian, Hispanic, and African populations. As global populations continue to age, the burden of GA is expected to increase, emphasizing its growing public health burden [4,5].

Risk Factors

Age is the most significant risk factor for the development of AMD, with the risk increasing 4.2-fold with each decade of life. A positive family history, particularly in first-degree relatives, is also associated with an elevated risk [10,11]. Modifiable risk factors such as smoking and obesity have been consistently linked to AMD [10,11]. Potential contributors are hypertension and cardiovascular disease, but their current association is less clearly established [10,11]. 

Pathophysiology

The development and progression of GA involve a complex and multifactorial process involving RPE dysfunction, oxidative stress, and chronic inflammation, with complement system activation playing a central role [13]. The mechanism underlying GA is not yet completely understood; however, a number of key mechanisms have been identified.

Under normal conditions, the RPE is responsible for maintaining retinal homeostasis and clearing metabolic waste. In GA, there is an RPE dysfunction that results in impaired phagocytosis of outer segment debris and the accumulation of metabolic waste. The latter contributes to drusen formation and compromises the structural integrity of the outer retina [13,14]. Drusen tend to deposit along Bruch's membrane, interfering with the interaction between Bruch's membrane and the RPE. This results in reduced nutrient and waste exchange, which contributes to hypoxia and retinal tissue degeneration [14-16].

Aging further exacerbates this process through increased oxidative stress. Advanced age leads to mitochondrial dysfunction, which causes reactive oxygen species (ROS) to accumulate and further exacerbate RPE and photoreceptor injury [15]. Tobacco smoking, hypercholesterolemia, and cardiovascular disease are environmental risk factors associated with increased ROS levels that also cause oxidative damage to the retina [5,15].

Additionally, drusen deposits and oxidative stress contribute to a chronic, low-grade inflammatory state and immune dysregulation, in which the complement system plays a key role. The overactivation of the complement cascade enhances the recruitment of inflammatory effectors and membrane attack complex (MAC) formation, both of which directly contribute to RPE and photoreceptor cell loss [4,15,16]. Collectively, these mechanisms explain the characteristic atrophy of GA and highlight key pathogenic processes that are targets for emerging therapeutic strategies. 

The complement system

General Description

The complement system plays a central role in the immune system, and it has been increasingly involved in the pathogenesis of GA. It consists of a series of enzymatic reactions, enabling rapid responses to invading microbes and cellular stress signals. It is usually classified into three main pathways: the classical, lectin, and alternative [17]. To maintain homeostasis and to ensure the complement system only targets foreign particles instead of healthy cells in the body, an appropriate balance between activation and inhibition of the system is necessary [17].

The classical pathway is triggered when complement component 1q (C1q) recognizes specific patterns on foreign microbes or damaged cells, while the lectin pathway is initiated when mannose-binding lectin (MBL), collectins, and ficolins bind mannose-containing polysaccharides on microorganisms. All these molecules act as pattern recognition receptors, and they bind pathogen surfaces or stressed cells and redirect complement activation to these sites [18].

The alternative pathway is more spontaneous. It starts with the hydrolysis of complement component C3, which then tags surfaces with C3b and activates the pathway. While the alternative pathway is continuously active at a low level, the classic and lectin pathways are only activated when the triggering molecule is present. The alternative pathway's specific function is to detect targets that might be undetected by the other two pathways [19].

The main convergence point of the three pathways is the cleavage of C3 to form C3a and C3b through C3 convertases. This corresponds to the central part of the complement cascade. Then, the formation of C5 convertase complexes is formed; C5 is cleaved by C5 convertases and turned into C5a and C5b. All these complement proteins, along with others like C4a, contribute to the release of histamine, tumor necrosis factor, and other factors, inducing inflammation, which recruits antibodies, complement, and phagocytic cells to the site. C5b initiates the formation of the MAC, which is responsible for final pathogen elimination [18,20].

The Role in GA 

When the complement cascade becomes overactive or poorly regulated, it can drive a self-perpetuating cycle of tissue damage and inflammation. This overactivation has been consistently implicated in the development and progression of GA [21].

Patients with intermediate AMD and late dry AMD have shown high levels of complement activation when compared to early AMD or patients with CNV. An accumulation of the MAC in histological eye sections from young donors has also been detected, which suggests that there is some level of complement activation that contributes to normal eye homeostasis [21]. However, with aging and conditions such as AMD, this deposition of the MAC increases in the choriocapillaris and Bruch's membrane and later, in advanced stages, in the RPE cells. This accumulation may contribute to endothelial damage and RPE degeneration [17,21].

Complement proteins such as C3, C5, complement factor H (CFH), CFI, CFD, and CFB have also been identified as contributors to AMD [22]. Complement activation contributes to drusen formation, and fragments of C5 and C3 have been detected within drusen of AMD patients [16,17]. Patients with AMD exhibit elevated serum levels of C3d, C3a, and C5a, reflecting increased activity of the alternative complement pathway. Altered plasma levels of CFD and CFI have also been reported [16].

Genetic variations in C3, CFH, and CFI also contribute to GA, by increasing complement activation or reducing regulatory inhibition, thus shifting the balance toward increased inflammatory activity. For instance, early genome-wide and sequencing analysis identified a variant in the CFH gene (CFH Y402H) that significantly increases AMD risk. Other AMD risk-associated polymorphisms in complement-related genes such as CFH, CFI, C3, and C2/CFB were also identified by the AMD Consortium [20]. Given its central role in disease progression, the complement cascade has become a major therapeutic target.

Treatment for GA

Although non-neovascular (dry) AMD represents for the majority of cases of AMD, accounting for 80-85% of them, therapeutic advances have predominantly focused on the neovascular (wet) form, which affects around 15-20% of patients [23]. Despite its lower prevalence, wet AMD is responsible for nearly 80% of severe vision loss associated with the disease [23]. This difference in clinical characteristics has directed research priorities over the past decades [23]. Therefore, the development of therapies for wet AMD has advanced rapidly, whereas treatment options for dry AMD and GA have remained limited [24]. The introduction of anti-vascular endothelial growth factor (anti-VEGF) agents transformed the management of wet AMD, successfully treating CNV. In contrast, interventions for dry AMD were limited to observational follow-up, vision rehabilitation, and vitamin formulations to prevent intermediate AMD from progressing into advanced AMD. All this evidence highlighted a significant need for novel therapies [24].

AREDS-Based Vitamin Formulations

The use of vitamin supplements for preventing progression to advanced AMD is attributed to clinical findings from the AREDS study. The AREDS and AREDS2 were multicenter phase III randomized clinical trials designed to evaluate nutritional supplementation on AMD progression [25]. In the AREDS, 4757 patients who had no AMD or unilateral late AMD received either placebo, antioxidants, zinc, or the combination [25]. In the AREDS2, 4203 patients with bilateral large drusen or unilateral late AMD received each supplement alone, or with additional lutein/zeaxanthin, or with additional docosahexaenoic acid and eicosapentaenoic acid, or the combination [25]. 

Evidence from the study showed there was a decrease in the risk of progression to advanced AMD in participants with intermediate AMD [26]. Nevertheless, this effect was more significant in avoiding progression to wet AMD than it was in preventing progression to dry AMD [26]. Moreover, evidence states this supplementation does not directly benefit patients with GA and does not significantly reduce the progression of GA or have any effect on progression toward the fovea, leaving patients with this condition without any real therapy at the time [8].

Eculizumab

The urgent need for effective GA therapies prompted multiple clinical investigations. However, many early trials failed to demonstrate a significant benefit. One example is the COMPLETE trial (ClinicalTrials.gov identifier NCT00935883), a phase II clinical trial that evaluated the effect of intravenous (IV) eculizumab, a systemic inhibitor of C5, on the growth of GA in a small subset of patients [27]. Its main outcomes did not reveal any benefit of eculizumab when compared with placebo [27].

Lampalizumab

Another exemplification is lampalizumab, an anti-complement factor D therapy [28]. The MAHALO phase II trial (ClinicalTrials.gov identifier NCT01229215) evaluated the use of intravitreal (IVT) lampalizumab in patients with GA without CNV [28]. Results of this trial showed the injection led to a significant reduction of GA progression in approximately 20%. This promising finding then encouraged the development of two other trials: CHROMA (ClinicalTrials.gov identifier NCT02247479) and SPECTRI (ClinicalTrials.gov identifier NCT02247531) [29,30]. They corresponded to two phase III randomized clinical trials testing the efficacy and safety of IVT lampalizumab in patients with bilateral GA and no CNV [29]. Despite the initial promising outcome of MAHALO, neither the CHROMA nor the SPECTRI trials showed a reduction in GA lesion area [29,30]. Of further concern, the patients experienced a significant decline in best-corrected visual acuity (BCVA) while receiving the treatment [29]. Other unsuccessful agents in this matter include NGM621, tesidolumab, and tandospirone [24]. While the primary endpoints of the trials were not met, the gathered data provided information that was not futile and was later utilized for improvement and refining protocols in subsequent studies [24].

Complement inhibition therapy 

**Figure 1:**
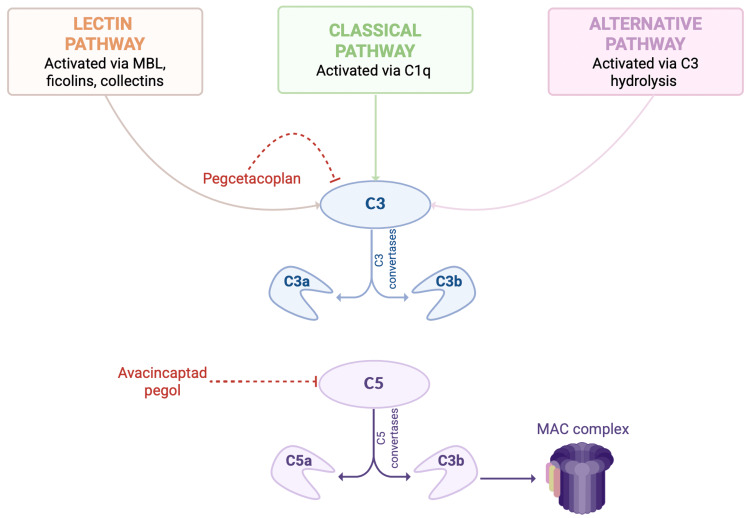
Pharmacological targets of pegcetacoplan and avacincaptad pegol. This simplified representation of the complement cascade illustrates that the three complement activation pathways converge at C3. Pegcetacoplan acts upstream in the cascade by inhibiting C3, whereas avacincaptad pegol inhibits C5 at a more downstream point. MBL: mannose-binding lectin; MAC: membrane attack complex Created with BioRender.com. This figure was manually assembled by the authors, and no artificial intelligence (AI) tools were used in its creation.

Encouragingly, two complement inhibition therapies demonstrated efficacy in slowing GA lesion progression and were finally approved by the FDA in 2023: IVT pegcetacoplan and avacincaptad pegol [9]. The therapeutic targets of these therapies involving the complement cascade are shown in Figure 1. 

Before this milestone, the approach to the disease was based on preventing intermediate AMD progression to advanced AMD with the AREDS2 vitamin formulation, clinical observation, and regular ophthalmology follow-ups [22,31]. Currently, several therapeutic targets are being evaluated, including complement proteins like C1q, C3, C5, CFB, CFH, CFI, and the MAC [16]. Additional candidate therapies targeting non-complement-mediated mechanisms are also being explored, including gene and cell-based therapies [13].

Pegcetacoplan (Syfovre™)

Pegcetacoplan is a pegylated peptide inhibitor of C3 cleavage, meaning it is formed by two copies of a tridecapeptide that are covalently conjugated to a linear polyethylene glycol (PEG) molecule [32]. It corresponds to the first FDA-approved complement inhibitor for GA secondary to AMD.

OAKS and DERBY clinical trials

This approval was based on the results from the OAKS (ClinicalTrials.gov identifier NCT03525613) and DERBY (ClinicalTrials.gov identifier NCT03525600) clinical trials. They were two 24-month phase III, randomized, double-masked studies that compared patients receiving IVT pegcetacoplan 15 mg/0.1 mL or sham treatment every month (EM) or every other month (EOM) [33].

Inclusion criteria for both studies contemplated patients of ≥60 years with GA secondary to AMD, with either foveal or subfoveal lesions, a total GA lesion area of 2.5-17.5 mm^2^ for unifocal lesions and ≥1.25 mm^2^ if multifocal, the presence of perilesional hyperautofluorescence in fundus autofluorescence, and a BCVA of ≥24 letters on the Early Treatment Diabetic Retinopathy Study (ETDRS) charts [33,34]. Key exclusion criteria included GA secondary to a condition other than AMD in either eye and CNV in the study eye (active or personal history of CNV) including RPE tear. CNV in the fellow eye was not an exclusion criterion. The primary endpoint of the trial was the change in the total area of GA lesions based on fundus autofluorescence [34].

Primary Endpoint Results in OAKS 

In the 12-month results, OAKS revealed a reduction in lesion growth area of 22% (p = 0.0003) with pegcetacoplan EM and 16% (p = 0.0052) with pegcetacoplan EOM when compared to sham, both with p-values representing statistical significance [35]. Therefore, the primary endpoint at 12 months was met. At 24 months, OAKS revealed that pegcetacoplan EM and EOM slowed the growth of GA lesions by 22% (p < 0.0001) and 18% (p = 0.0002), respectively, compared with sham [34,36]. 

Primary Endpoint Results in DERBY 

The 12-month results from DERBY showed a reduction in lesion growth area of 12% (p = 0.0528) with EM treatment and 11% (p = 0.0750) with EOM treatment, but data didn't reach statistical significance [35]. Therefore, the primary endpoint at 12 months was not met in DERBY [35]. At 24 months, DERBY showed a 19% (p = 0.0004) and 16% (p = 0.0030) lesion growth reduction, with pegcetacoplan EM and EOM, respectively, now with statistical significance [34,36]. 

Primary Endpoint Results in OAKS and DERBY Combined Studies

The combined studies data from OAKS and DERBY demonstrated that the growth difference in atrophic lesions was 16% (p = 0.0001) with EM treatment and 14% (p = 0.0014) with EOM treatment when compared to sham, at 12 months. At 24 months, the difference was of 21% (p < 0.0001) and 17% (p < 0.0001) with EM and EOM pegcetacoplan when compared to sham, respectively [36].

On the other hand, analysis of reductions in GA lesion growth by lesion location included patients with subfoveal and nonsubfoveal lesions. It showed that patients in the nonsubfoveal GA subgroup had a 26% (p < 0.0001) reduction of lesion growth with EM treatment and 22% (p < 0.0001) reduction with EOM treatment when compared to sham, at month 24. Patients with subfoveal GA showed a reduction of 19% (p < 0.0001) and 16% (p = 0.0003) with pegcetacoplan EM and EOM when compared to sham, at month 24 [34,36]. 

Secondary Endpoint Results in OAKS and DERBY Combined Studies 

In the combined study results, secondary endpoints, including BCVA, maximum reading speed, and functional reading independence index, showed no statistically significant differences between the treatment and sham groups at 24 months [34,36]. Specifically, the EM group showed a mean BCVA reduction of 0.946 letters compared with the sham group. However, this difference was not statistically significant (p = 0.3558). The EOM group showed a mean BCVA reduction of 1.890 letters compared with the sham group, which also did not reach statistical significance (p = 0.069). 

Adverse Effects in OAKS and DERBY

In these studies, the adverse events most often reported were wet AMD, ocular discomfort, vitreous floaters, and conjunctival hemorrhage, each observed in more than 5% of patients. Complications like endophthalmitis, retinal detachment, and elevated intraocular pressure usually associated with IVT injections were present but reported in less than 1% of the participants [37]. The general safety profile for pegcetacoplan is similar to that of other IVT agents, except for the new-onset wet AMD, for which the incidence is higher in patients receiving treatment [34]. Importantly, there were three documented serious cases of optic neuropathy, with the use of pegcetacoplan, throughout the 24 months [38].

GALE clinical trial

To investigate the long-term efficacy and safety of IVT pegcetacoplan 15 mg/0.1 mL, a GALE (ClinicalTrials.gov identifier NCT04770545) extension study was developed: a 36-month, phase III, multicenter, open-label extension study [39]. Inclusion criteria for participants in GALE were the same as for the OAKS/DERBY, which were previously described [39]. Nearly 80% of the enrolled patients were participants of the OAKS and DERBY trials, and the rest were from the APL2-103 study [39]. In GALE, patients who received sham previously now change to receiving IVT pegcetacoplan. The primary endpoint for this study was the incidence and severity of ocular and non-ocular treatment-emergent adverse effects (TEAEs) [39]. Secondary endpoints included changes from baseline in total GA area and mean rate of change of GA [39].

Primary Endpoint Results in GALE

At month 12, GALE showed that the most common non-ocular TEAE was COVID-19, occurring in 13% of participants, as the study was conducted during the COVID-19 pandemic [40]. Moreover, the trial demonstrated that the most common ocular TEAEs varied between treatment groups [40]. Patients who received 36 months of EM and EOM injections are the EM-EM group and EOM-EOM groups, respectively. The ones who transitioned from sham to injections are the sham-EM and sham-EOM groups. 

Regarding the ocular TEAEs, new-onset AMD occurred in 7.9% of patients in the EM-EM group, and increased intraocular pressure (IOP) was reported in 5.2% in the EOM-EOM group [40]. Vitreous floaters were reported in 10% of participants in the sham-EM group and ocular discomfort in 7% in the sham-EOM group [40]. No cases of occlusive or non-occlusive vasculitis or retinitis were reported during the first 12 months of the GALE study [40].

Secondary Endpoint Results in GALE

Since all patients in GALE are now receiving active treatment instead of sham injections, the sham treatment group data was projected with a thorough method, so comparisons between treatment patients and sham patients could be made [7]. Regarding secondary endpoints, GALE demonstrated that the overall population in the EM-EM group and the EOM-EOM group had a 25% and 20% reduction of GA lesion area (both with p<0.0001) when compared to the projected sham treatment results at 36 months (month 0 to month 36) [7,40]. 

The results were also classified considering lesion location. Patients with subfoveal GA lesions showed a 21% (p < 0.0001) and 19% (p = 0.0001) reduction of the growth of the GA lesion with the EM-EM and EOM-EOM injections, respectively, when compared to projected sham at 36 months (month 0 to month 36) [40]. In nonsubfoveal lesions, the percentages were 32% (p < 0.0001) and 26% (p = 0.0002) reduction with EM-EM and EOM-EOM groups when compared to projected sham at 36 months [40].

Avacincaptad pegol (Izervay™) 

Avacincaptad pegol is an aptamer, a short RNA sequence, that inhibits C5 cleavage, thereby impeding the formation of C5a and C5b and subsequent formation of the MAC [41]. This mechanism has the potential to reduce complement-mediated retinal cell damage [41]. Similar to pegcetacoplan, clinical trials have been conducted to test this agent's efficacy and safety.

GATHER1 clinical trial

The GATHER1 study (ClinicalTrials.gov identifier NCT02686658) was a 12-month, prospective, randomized, double-masked phase II/III study. It evaluated changes in GA lesion progression with the IVT administration of avacincaptad pegol versus sham injections [10]. The trial was divided into two parts: the first part randomized patients to receive either 1 mg or 2 mg of avacincaptad pegol or sham EM, while subjects in the second part received either 2 mg or 4 mg (in two separate 2 mg injections) of avacincaptad pegol or sham EM. Only doses of 2 mg and 4 mg were included for statistical analysis [10,41].

Inclusion criteria for patients included age ≥50 years, with BCVA between 20/25 and 20/320 in the study eye and having nonsubfoveal GA secondary to AMD and in part within 1500 μm from the foveal center. The lesion was included if the total GA area was between 2.5 and 17.5 mm^2^ by autofluorescence, or if multifocal, at least one lesion had to be ≥1.25 mm^2^. All eyes with CNV and diabetic retinopathy were excluded. The primary efficacy endpoint was the mean rate of change in GA area, and secondary endpoints were the mean change in BCVA and in low-luminance visual acuity (LLVA) from baseline [42].

Primary Endpoint Results in GATHER1

In the GATHER1 12-month results, the 2 mg group showed a reduction of 27.4% (p = 0.0072) in GA lesion growth rate, while the 4 mg group showed a 27.8% (p = 0.0051) reduction when compared to sham [35,42]. At the 18-month follow-up, there were a 28.1% and a 30% reduction of lesion growth rate in the 2 mg and 4 mg groups, respectively, when compared to sham [43,44].

GATHER2 clinical trial

Later on, GATHER2 (ClinicalTrials.gov identifier NCT04435366) was conducted based on the optimistic results of GATHER1 [43]. GATHER2 was a 24-month phase III, randomized, double-masked trial that investigated the effects of 2 mg of avacincaptad pegol or sham EM in GA for the first 12 months and then 2 mg of avacincaptad pegol EM or EOM or sham. Eligible patients had the same characteristics as patients enrolled in GATHER1. The primary endpoint was GA lesion size measured by fundus autofluorescence, and secondary endpoints were the same as previously described for GATHER1 [44].

Primary Endpoint Results in GATHER2

The 12-month results of GATHER2 revealed that the rate of GA growth was reduced by 14.3% with 2 mg avacincaptad pegol EM when compared to sham [43]. The 24-month results revealed that patients with 2 mg EM had a 14% (p = 0.0165) reduction and 2 mg EOM represented a 19% (p = 0.0015) reduction when compared to sham treatment [43,45]. 

Secondary Endpoint Results in GATHER2

At 12 months, no meaningful differences in BCVA or LLVA between the group receiving treatment and sham were observed in the mean change from baseline [44]. At 24 months, BCVA or LLVA remained similar to the one-year results, with no real difference in these parameters with the treatment when compared to sham [45].

Adverse Effects in GATHER1 and GATHER2

In the GATHER1 and GATHER2 trials, ocular adverse events included conjunctival hyperemia, conjunctival edema, punctate keratitis, cataracts, vitreous detachment, CNV, eye pain, and increased IOP [38]. A real-world experience safety profile reported an increased risk of conversion to exudative AMD and an increase in IOP after avacincaptad pegol injection. There was no intraocular inflammation, ischemic optic neuropathy, or endophthalmitis. However, the mean follow-up time for this real-world study was only 120 days, which limits the assessment of long-term safety data [46].

Table 1 summarizes the important information presented in this review about the DERBY, OAKS, GALE, GATHER1, and GATHER2 studies.

**Table 1:** Key clinical trials evaluating pegcetacoplan and avacincaptad pegol in GA. IVT: intravitreal; GA: geographic atrophy; EM: every month; EOM: every other month

Clinical trial (identifier)	Study design	Medication	Complement target	Route	Dosing regimen	Primary outcome (GA lesion growth)
OAKS (NCT03525613)	Phase III, randomized, double-masked	Pegcetacoplan	C3 inhibition	IVT	15 mg/0.1 mL or sham EM or EOM	12 months: 22% reduction with EM and 16% reduction with EOM. 24 months: 22% reduction with EM and 18% reduction with EOM
DERBY (NCT03525600)	Phase III, randomized, double-masked	Pegcetacoplan	C3 inhibition	IVT	15 mg/0.1 mL sham EM or EOM	12 months: no statistically significant reduction. 24 months: 19% reduction with EM and 16% reduction with EOM
GALE (NCT04770545)	Open-label extension study	Pegcetacoplan	C3 inhibition	IVT	15 mg/0.1 mL EM or EOM	36 months: 25% reduction with EM and 20% reduction with EOM compared with the projected sham
GATHER1 (NCT02686658)	Phase II/III, randomized, double-masked	Avacincaptad pegol	C5 inhibition	IVT	1 mg or 2 mg or sham EM and 2 mg or 4mg or sham EM	12 months: 27.4% reduction (2 mg) and 27.8% reduction (4 mg). 18 months: 28.1% reduction (2 mg) and 30% reduction (4 mg)
GATHER2 (NCT04435366)	Phase III, randomized, double-masked	Avacincaptad pegol	C5 inhibition	IVT	First 12 months: 2 mg or sham EM; second 12 months: 2 mg or sham EM or EOM	12 months: 14.3% reduction with 2 mg EM. 24 months: 14% reduction with EM and 19% reduction with EOM

Discussion and possible future directions

Both pegcetacoplan and avacincaptad pegol have demonstrated efficacy in slowing the rate of GA lesion growth compared with sham treatment in major clinical trials, including OAKS, DERBY, GALE, GATHER1, and GATHER2. These findings are further supported by a Cochrane meta-analysis, which concluded that pegcetacoplan administered as a 15 mg IVT injection either EM or EOM reduces GA lesion growth in both subfoveal and nonsubfoveal GA [47].

An important observation arises from the differences between the OAKS and DERBY trials. While OAKS met its primary endpoint at 12 months, DERBY did not initially demonstrate statistical significance. This discrepancy may be partially explained by differences in baseline patient characteristics, including variations in lesion distribution and the proportion of patients with subfoveal involvement. Nevertheless, both trials achieved statistical significance at 24 months, suggesting that the therapeutic effect of complement inhibition may become more apparent over longer follow-up periods [34].

Regarding avacincaptad pegol, early evidence from GATHER1 demonstrated a meaningful reduction in GA lesion growth, although the Cochrane meta-analysis expressed some uncertainty because of the limited amount of available data [47]. More recent results from GATHER2 have further supported the ability of 2 mg avacincaptad pegol to reduce lesion progression compared with sham treatment [45].

Direct comparisons between pegcetacoplan and avacincaptad pegol remain challenging because of important differences in study design and patient populations across clinical trials. Studies evaluating pegcetacoplan included patients with both nonsubfoveal and subfoveal GA lesions, whereas studies evaluating avacincaptad pegol primarily enrolled patients with nonsubfoveal GA lesions. Indirect analyses comparing nonsubfoveal lesion subgroups have suggested potentially greater reductions in GA lesion growth with pegcetacoplan relative to avacincaptad pegol [48]. However, cross-trial comparisons are inherently limited by differences in eligibility criteria, baseline disease characteristics, outcome measurements, and follow-up protocols. Consequently, these observations should be interpreted with caution and considered hypothesis-generating rather than conclusive evidence of superior efficacy. Although broader upstream inhibition of the complement cascade at the C3 level has been proposed as a possible explanation for the differential therapeutic effects [48], direct head-to-head comparative studies are required before definitive conclusions regarding relative efficacy can be established.

Despite the consistent reduction in lesion growth observed across clinical trials, improvements in functional outcomes such as BCVA and LLVA have remained limited, and studies have consistently failed to demonstrate clinically meaningful visual benefit. This highlights an important challenge in the interpretation of structural outcomes in GA, as anatomical slowing of lesion progression does not necessarily translate into measurable improvements in visual function or patient quality of life within the follow-up periods evaluated in current studies.

Functional endpoints may vary considerably between patients and may not adequately capture subtle or progressive visual impairment in GA [45,49]. Additionally, in some patients, foveal involvement may occur relatively late in the disease course, potentially limiting detectable changes in visual acuity despite ongoing lesion enlargement [50]. Consequently, many clinical trials rely primarily on structural measurements as primary endpoints. Although these anatomical measurements remain valuable and reproducible for monitoring disease progression, they represent surrogate endpoints whose direct correlation with meaningful functional outcomes remains uncertain and raises important questions regarding the real-world clinical significance of complement inhibition therapies.

One possible explanation for the discrepancy between anatomical and functional outcomes is that visual impairment in GA may depend not only on lesion size but also on lesion characteristics and retinal location, which can variably affect reading speed and visual acuity despite similar rates of GA expansion [49]. Furthermore, fundus autofluorescence, which is commonly used to quantify GA lesion progression, may itself be influenced by the presence of neovascularization, potentially confounding the interpretation of structural outcomes [49]. In addition, GA has a slow and progressive natural history, which may require longer follow-up periods before potential functional benefits become clinically detectable [45,49]. Future studies incorporating patient-centered functional measures, including reading performance, contrast sensitivity, and vision-related quality-of-life assessments, may provide a more comprehensive evaluation of therapeutic benefit in GA.

Clinical trial data suggest that pegcetacoplan and avacincaptad pegol are generally well tolerated; however, emerging post-marketing and real-world safety data have raised important concerns regarding ocular adverse events associated with these therapies. Among the most clinically relevant complications is an increased incidence of conversion to neovascular AMD observed in treated eyes, which may partially result from alterations in choriocapillaris homeostasis and endothelial regulation following complement blockade [9].

In addition, post-marketing pharmacovigilance analyses have identified rare but potentially vision-threatening adverse events, including intraocular inflammation, retinal vasculitis, hemorrhagic occlusive retinal vasculitis, ischemic optic neuropathy, and endophthalmitis, particularly in association with pegcetacoplan use [38]. Although these findings are derived primarily from spontaneous reporting databases and cannot establish definitive causality, they highlight the importance of continued long-term safety surveillance and careful patient monitoring during treatment initiation and follow-up.

Interpretation of currently available real-world safety data should also be approached cautiously, as reporting systems such as the FDA Adverse Event Reporting System (FAERS) are subject to underreporting, incomplete case characterization, variable reporting practices, and inability to determine true incidence rates or causal relationships [38]. Additionally, differences in market availability and cumulative clinical exposure between pegcetacoplan and avacincaptad pegol may partially influence the apparent frequency of reported adverse events [38].

Treatment with complement inhibitors involves chronic IVT administration, which carries procedural risks inherent to repeated intraocular injections, including endophthalmitis and other injection-related complications that may potentially compromise vision [50]. The adverse event profile associated with these therapies must also be carefully considered, as ocular inflammation and the development of neovascular complications may necessitate additional invasive interventions and further increase treatment burden [50].

Economic considerations and treatment adherence also represent important clinical challenges in the long-term management of GA. Based on 2022 Medicare reimbursement data, the estimated two-year cost of pegcetacoplan therapy was approximately $70,000 for EM injections and $34,600 for EOM treatment regimens [51]. Similarly, the estimated cost of avacincaptad pegol treatment over a two-year period was approximately $67,400 and $40,600 for EM and EOM regimens, respectively [52]. Long-term patient adherence may therefore be compromised by the chronic and invasive nature of treatment, in addition to the substantial economic burden associated with these therapies. Consequently, given the modest functional benefits observed, treatment decisions should involve individualized risk-benefit assessment and shared decision-making between physicians and patients.

The lack of approval of pegcetacoplan by the European Medicines Agency (EMA) may reflect ongoing concerns regarding limited functional benefit, uncertainty surrounding long-term safety, and an unfavorable cost-effectiveness profile, particularly in the context of chronic treatment burden and modest anatomical outcomes. In its assessment, the EMA concluded that the magnitude of pegcetacoplan's benefit did not sufficiently outweigh the potential risks and uncertainties associated with therapy [53].

At present, complement inhibitors remain the only FDA-approved pharmacologic therapies for GA, yet there is still no clear consensus regarding their optimal use in clinical practice. Although these therapies represent a significant advancement in the management of the disease, additional long-term data are needed to better characterize their impact on visual function, quality of life, and long-term safety outcomes in real-world settings.

Future research may benefit from improving patient selection through the development of predictive models capable of identifying patients most likely to benefit from therapy. Additional real-world evidence and long-term observational studies will also be essential to better characterize treatment effectiveness and safety profiles over time. Emerging therapeutic strategies continue to be actively investigated. Gene therapy approaches aim to provide the sustained inhibition of complement activation, while cell-based therapies seek to restore or replace damaged retinal cells [54]. Therapies such as JNJ-1887, which delivers a soluble form of CD59 to inhibit MAC formation, represent promising avenues for future intervention [55,56]. Similarly, cell-based therapies including RG6501 (OpRegen), ASP7317, and Eyecyte-RPE are currently under investigation [56].

Overall, complement inhibition has marked a significant advancement in the management of GA. However, continued research remains necessary to optimize treatment strategies, better define patient selection, clarify long-term safety profiles, and determine whether anatomical benefits ultimately translate into meaningful functional improvements and better quality of life for patients.

## Conclusions

GA remains a major cause of irreversible vision loss, with limited therapeutic options until recently. The development of complement inhibitors represents a significant advancement, as both pegcetacoplan and avacincaptad pegol have demonstrated the ability to slow the progression of GA lesions. However, their impact on functional outcomes such as visual acuity remains limited, and the side effect profile still requires longer follow-ups and strict monitoring.

While these therapies mark an important step in the management of GA, several challenges remain, among them optimal patient selection, treatment dosage, and long-term efficacy. Every patient should be assessed individually, always prioritizing their best interest and comprehensive management. Ongoing research and emerging therapeutic strategies are warranted to improve the range of available options for patients with GA.
